# A CpG-adjuvanted intranasal enterovirus 71 vaccine elicits mucosal and systemic immune responses and protects human SCARB2-transgenic mice against lethal challenge

**DOI:** 10.1038/s41598-018-28281-5

**Published:** 2018-07-16

**Authors:** Yu-Li Lin, Yen-Hung Chow, Li-Min Huang, Szu-Min Hsieh, Pei-Yun Cheng, Kai-Chieh Hu, Bor-Luen Chiang

**Affiliations:** 1 Department of Medical Research, National Taiwan University Hospital, Taipei, Taiwan; 2 National Institute of Infectious Diseases and Vaccinology, National Health Research Institutes, Zhunan Town, Miaoli County, Taiwan; 3 Graduate Institute of Biomedical Sciences, China Medical University, Taichung, Taiwan; 4 Department of Pediatrics, National Taiwan University Hospital, Taipei, Taiwan; 5 Internal Medicine, National Taiwan University Hospital, Taipei, Taiwan

## Abstract

Enterovirus 71 (EV71) is an aetiological agent responsible for seasonal epidemics of hand-foot-and-mouth disease, which causes considerable mortality among young children. Mucosal vaccines can efficiently induce secretory IgA at mucosal surfaces and thereby prevent or limit infection at the site of virus entry. CpG oligodeoxynucleotides (ODNs), which resemble bacterial DNA, can induce the innate immune response through activation of Toll-like receptor 9. Here, we used CpG ODNs as adjuvants to investigate an EV71 mucosal vaccine in mice. In the EV71 + CpG group, the EV71-specific IgG and IgA titres in the serum, nasal wash, bronchoalveolar lavage fluid, and faeces were substantially higher than those in the EV71- and phosphate-buffered saline-treated groups. Moreover, the number of EV71-specific IgG- and IgA-producing cells was also higher in the EV71 + CpG group. Furthermore, T-cell proliferative responses and interleukin-17 secretion were markedly increased when CpG-adjuvanted EV71 was delivered intranasally. More importantly, the induced antibodies neutralised infection by EV71 of the C2 genotype and crossneutralised infection by EV71 of the B4 and B5 genotypes. Lastly, human scavenger receptor class B, member 2-transgenic mice intranasally immunised with the CpG-adjuvanted EV71 vaccine resisted a subsequent lethal challenge with EV71, indicating that CpG was an effective intranasal adjuvant for EV71 mucosal-vaccine development.

## Introduction

Enterovirus type 71 (EV71) is a small, nonenveloped, single-stranded RNA virus belonging to the human enterovirus A species of the family *Picornaviridae*^1–3^. EV71 causes seasonal epidemics of hand-foot-and-mouth disease (HFMD), aseptic meningitis, poliomyelitis-like paralysis, and acute brainstem encephalitis and is associated with severe and fatal neurological complications in infants and young children during the spring to fall seasons^4–6^. EV71 has emerged as a neuron-invasive virus responsible for several outbreaks in the Asia-Pacific region. In 1998, a major epidemic of EV71 in Taiwan caused 1.5 million infections, 405 with severe neurological complications, and resulted in 78 deaths in paediatric patients^7^. In China, more than 7 million HFMD cases and 2457 deaths were reported during EV71 epidemics occurring between 2008 and 2012^8^. Moreover, EV71 epidemics have recently been reported in the United States of America and in European countries^7^. EV71 can potentially infect the central nervous system (CNS), and a vaccine that is effective against EV71 could prevent virus-induced morbidity and mortality.

Currently, no antiviral drugs are available for successfully neutralising EV71 infection. For preventing EV71 outbreaks, the most effective approach would be to develop vaccines against EV71; accordingly, several strategies have been applied to develop various EV71 vaccines, including attenuated live-virus vaccines, inactivated-virus vaccines, virus-like-particle vaccines, and VP1-subunit or DNA vaccines^9–11^. Recently, two alum-adjuvanted inactivated-EV71 vaccines developed in mainland China showed favourable immunogenicity persistence and acceptable safety profiles in clinical trials, and these two EV71 vaccines have been approved for marketing in China^12^. However, the two vaccines were injected intramuscularly (IM), which is less acceptable for children than for adults. In this study, our aim was to investigate a safe, needle-free EV71 vaccine for young children, specifically a vaccine for mucosal delivery. In efforts to enhance immunity against infection at mucosal tissue barriers, the mucosal delivery of vaccine antigens can facilitate the development of improved vaccines; mucosal vaccines can efficiently induce secretory IgA at mucosal surfaces and thereby prevent or limit infection at the site of enterovirus entry^13^. However, to date, few studies have investigated the mucosal delivery of EV71 vaccines. In vaccine development, adjuvants are frequently used to augment the effects of vaccines by stimulating the immune system to respond more vigorously (than in the absence of adjuvants) and thus provide increased immunity to disease. Nasal inoculation with appropriate adjuvants is generating considerable research interest because this approach has the potential to establish robust mucosal immune responses while causing little pain or resistance compared with injections, particularly in children and newborns^14^. An optimal mucosal adjuvant would increase the secretion of mucosal antibodies and the residence time of antigens, while concurrently reducing the dose of antigen required.

Toll-like receptor (TLR) ligands can act as both systematic and mucosal adjuvants. Thus, CpG oligodeoxynucleotides (ODNs), which resemble bacterial DNA, were found to induce the innate immune response through the activation of TLR9, which is expressed exclusively on human B cells and plasmacytoid dendritic cells (pDCs); consequently, Th1-type-dominated immune responses were induced^15^. Three types of stimulatory CpG ODNs have been identified (types A, B, and C) based on their specific sequences and effects. Type A stimulates interferon (IFN)-α production in pDCs, whereas type B strongly activates B cells^16^. Type C features a complete phosphorothioate backbone harbouring CpG dinucleotides, and because the phosphorothioate linkage can reduce the sensitivity of CpG to nuclease digestion, the adjuvant activity is increased^17,18^. Type C CpG ODNs potently induce IFN-α secretion from pDCs and strongly activate B cells^19,20^. Moreover, *in vivo* studies have demonstrated that type C CpG ODNs combine the effects of type A and B ODNs^21^. Furthermore, CpG ODNs activate TLR9 in a species-specific manner. ODN2395 is a type C CpG ODN with a preference for human and murine TLR9^19^.

Here, we investigated the use of ODN2395 as an adjuvant for an EV71 mucosal vaccine. Mucosal immunisation with inactivated EV71 in formulation with this CpG ODN (hereafter referred to as CpG) effectively induced broad-spectrum immune responses; thus, this CpG-adjuvanted vaccine may represent a promising mucosal-vaccine candidate for preventing EV71 infection.

## Results

### CpG used as a nasal adjuvant induced systemic and mucosal EV71-specific antibody responses

**Figure 1:**
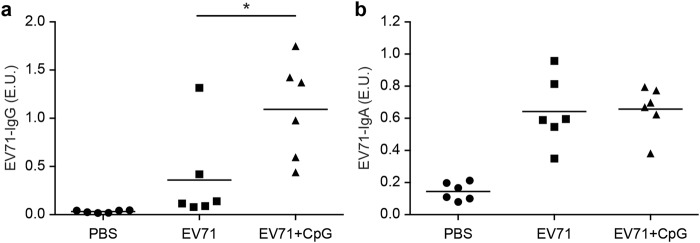
EV71-specific antibody responses measured in serum from BALB/c mice immunised with CpG as an adjuvant for a formalin-inactivated EV71 vaccine. Mice were intranasally immunised with PBS, formalin-inactivated EV71 (5 μg/dose), or formalin-inactivated EV71 (5 μg/dose) plus CpG (20 μg/dose) three times at 3-week intervals. ELISA was used to measure EV71-specific IgG (**a**) and EV71-specific IgA (**b**) in the serum of mice at 2 weeks after the third immunisation. Values shown are means ± SEMs of six mice in each experimental group; *p < 0.05. One representative study of three with similar results is shown. E.U.: ELISA units.

**Figure 2:**
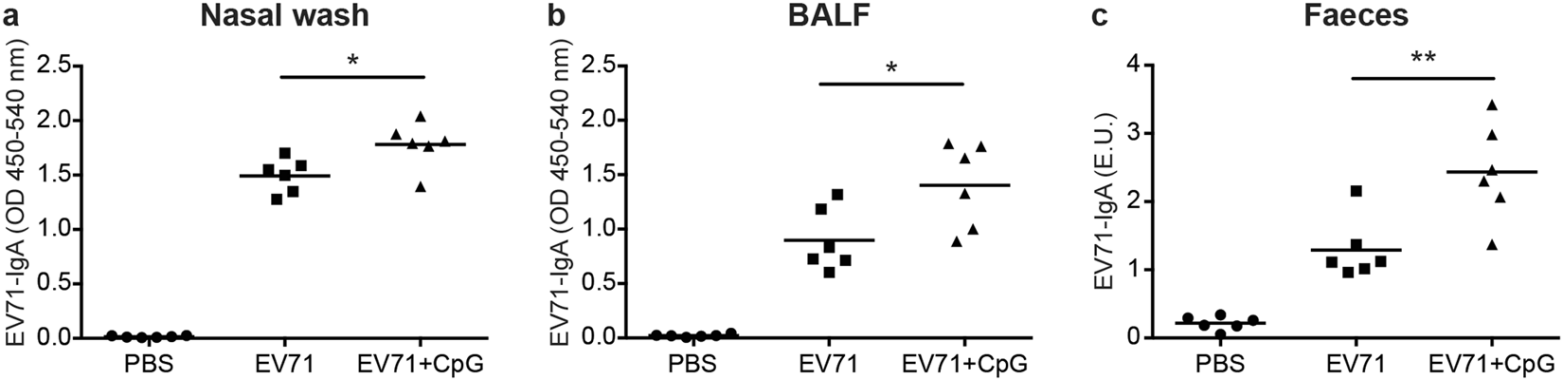
EV71-specific IgA titres in nasal wash, BALF, and faeces. Mice were intranasally immunised with PBS, formalin-inactivated EV71 (5 μg/dose), or formalin-inactivated EV71 (5 μg/dose) plus CpG (20 μg/dose) three times at 3-week intervals. ELISA was used to measure EV71-specific IgA in the nasal wash (**a**), BALF (**b**), and faeces (**c**) of mice at 2 weeks after the third immunisation. Values shown are means ± SEMs of six mice in each experimental group; *p < 0.05, **p < 0.01. One representative study of three with similar results is shown. BALF: bronchoalveolar lavage fluid.

We first examined whether CpG as a nasal adjuvant could enhance EV71-specific immune responses. Mice were nasally immunised three times at 3-week intervals with phosphate-buffered saline (PBS; mock), 5 μg formalin-inactivated EV71, or 5 μg formalin-inactivated EV71 adjuvanted with 20 μg CpG. Formalin-inactivated EV71 served as the positive control, and EV71-specific IgGs and IgAs in serum samples were detected using enzyme-linked immunosorbent assays (ELISAs). Compared with the PBS group, the EV71 and EV71 + CpG groups generated substantial amounts of EV71-specific IgG and IgA after the third vaccination (Fig. 1a,b), and notably, the serum titre of the EV71-specific IgG in the EV71 + CpG group was significantly higher than that in the EV71 group (*p* < 0.05; Fig. 1a) after the third immunisation. Furthermore, the EV71 and EV71 + CpG groups also generated considerable amounts of EV71-specific IgA in the nasal wash, bronchoalveolar lavage fluid (BALF), and faeces after the third vaccination (Fig. 2a–c). Moreover, the titre of the EV71-specific IgA in the EV71 + CpG group was significantly higher than that in the EV71 group in the nasal wash (*p* < 0.05), BALF (*p* < 0.05), and faeces (*p* < 0.01) after the third immunisation (Fig. 2a).

**Figure 3:**
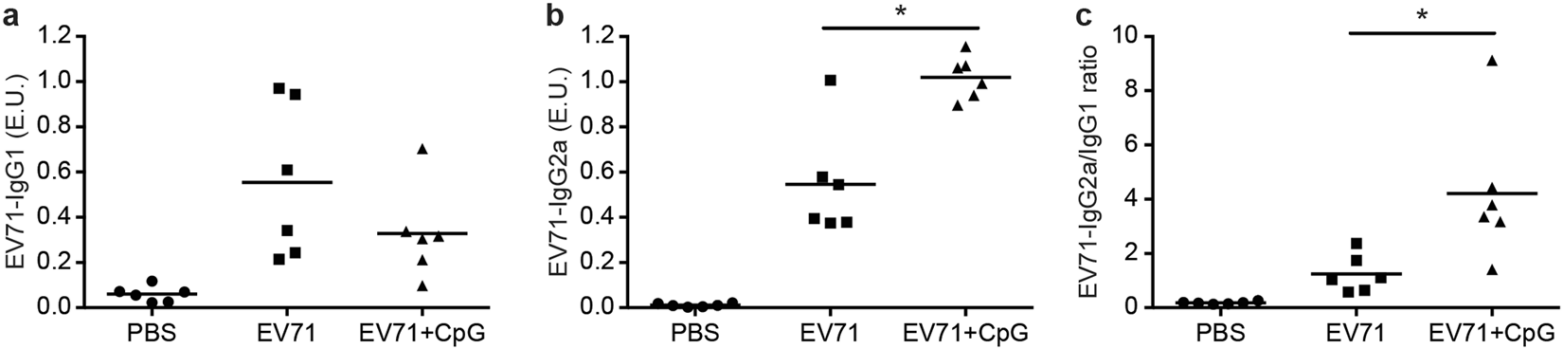
EV71-specific IgG1 and IgG2a responses in the serum. Effects of CpG as an adjuvant on EV71-specific IgG1 (**a**), EV71-specific IgG2a (**b**), and EV71-specific IgG2a-to-IgG1 ratio (**c**) in the serum of BALB/c mice at 2 weeks after the third immunisation with PBS or with formalin-inactivated EV71 alone or with CpG. Values shown are means ± SEMs of six mice in each experimental group; *p < 0.05. One representative study of three with similar results is shown.

Because adjuvants can also potentially influence the type of helper T-cell responses, we analysed EV71-specific IgG1 and IgG2a in the serum (Fig. 3). In mice vaccinated with EV71 plus CpG as adjuvants, the EV71-specific IgG2a production was significantly higher (*p* < 0.05) than that in mice immunised with EV71 alone (Fig. 3b), and the EV71-specific IgG showed an isotype bias towards the Th1 response (Fig. 3c). Overall, our results suggested that CpG used as a nasal adjuvant could effectively enable the induction of an EV71-specific systemic and mucosal immune response.

### Neutralisation and cross-reactivity

**Figure 4:**
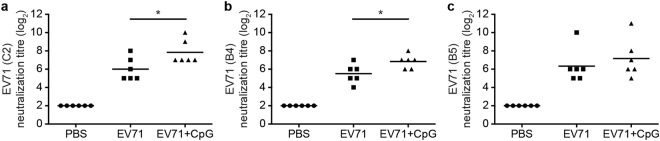
Neutralisation titres of serum antibodies against distinct EV71 strains. (**a**) TW2272/98 (C2 genogroup), (**b**) 200307025 (B4 genogroup), and (**c**) 20080738 (B5 genogroup). Sera were collected from mice at 2 weeks after the third immunisation with PBS or formalin-inactivated EV71 alone or with CpG and were serially diluted (2^3^–2^12^), mixed with the EV71 virus (strain TW2272/98, 200307025, or 20080738), and used to infect RD cells. After 4 days, the neutralisation titre was read as the highest dilution that resulted in the virus producing no cytopathic effect. Values shown are means ± SEMs of six mice in each experimental group; *p < 0.05. One representative study of three with similar results is shown. RD cells: rhabdomyosarcoma cells.

To determine whether the CpG-adjuvanted EV71 vaccine could elicit functional anti-EV71 antibodies, we used rhabdomyosarcoma cells (RD) cells and measured the titres of neutralising antibodies in the collected serum. Because the EV71 vaccine was derived from the TW2272/98 strain (C2 genogroup), which circulated in 1998, the neutralising-antibody titre was determined as the highest dilution that neutralised 100-fold 50% tissue-culture infectious dose (TCID_50_) of the TW2272/98 virus and resulted in the absence of any cytopathic effect on EV71-sensitive RD cells. All mice vaccinated with EV71 alone or with CpG as an adjuvant generated antibodies capable of neutralising EV71 of the C2 genotype (Fig. 4a), but the neutralising-antibody titre was significantly higher in mice vaccinated with CpG-adjuvanted EV71 than in mice vaccinated with EV71 alone (*p* < 0.05).

To investigate the cross-reactivity of the induced antibodies, the collected serum samples were tested in neutralisation assays performed using two other EV71 strains: 200307025, B4 genogroup, circulating in 2003 (Fig. 4b); and 20080738, B5 genogroup, isolated in 2008 (Fig. 4c). The neutralisation titres measured against the B4 genogroup were close to those against the C2 genogroup. Overall, these results demonstrated that specific antibodies capable of cross-neutralising distinct genogroups of EV71 were generated in the EV71-vaccinated mice.

### Effects of the CpG adjuvant on antibody-secreting B cells in EV71-vaccinated mice

**Figure 5:**
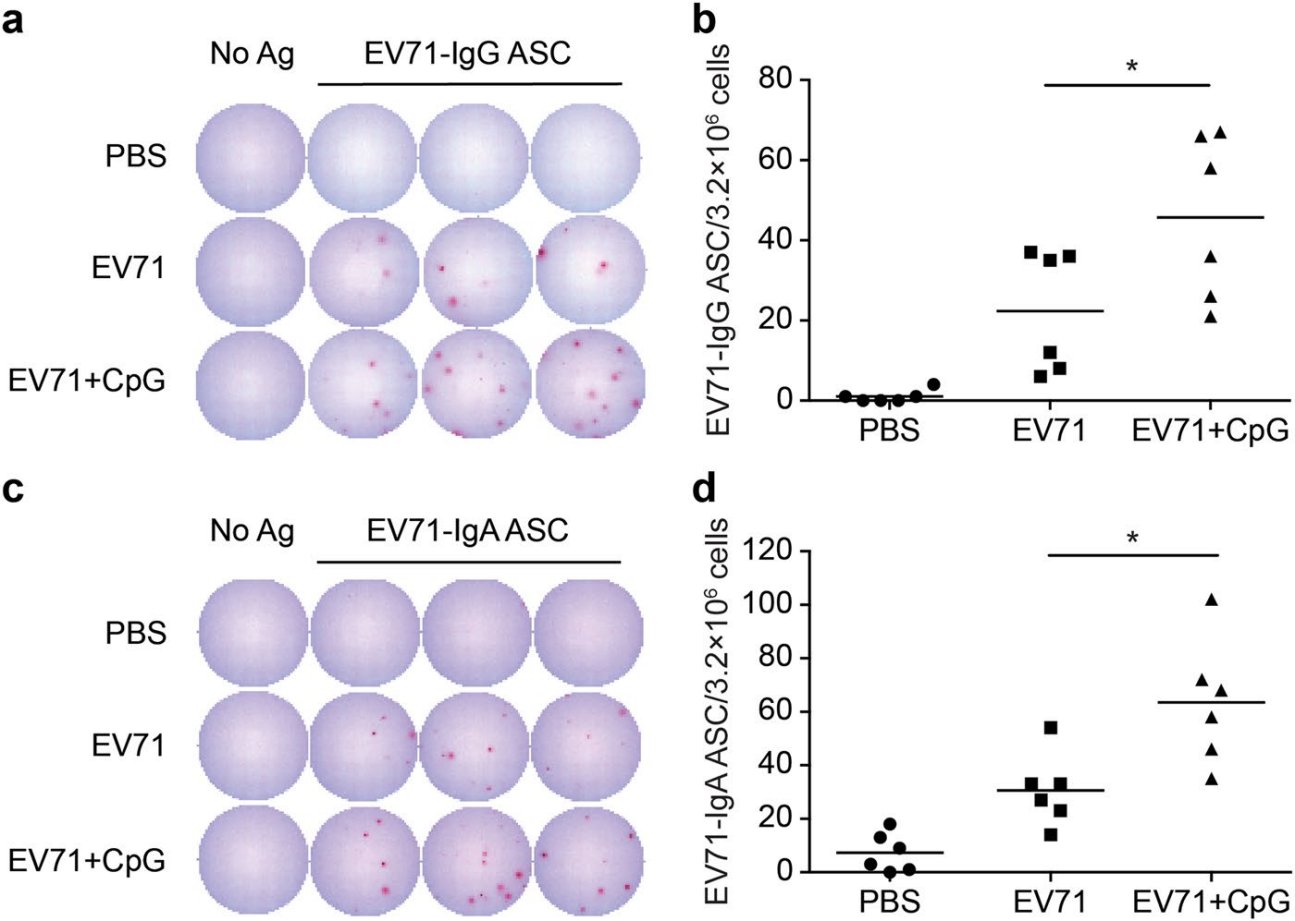
EV71-specific IgG antibody-secreting cells (ASCs) and IgA ASCs in mouse spleens. Mice were intranasally immunised with PBS or formalin-inactivated EV71 without/with CpG three times at 3-week intervals. Spleens were isolated at 2 weeks after the third immunisation, and the collected samples were used in EV71-specific ELISPOT assays to determine the numbers of IgG ASCs (**a**,**b**) and IgA ASCs (**c**,**d**). Values shown are means ± SEMs of six mice in each experimental group; *p < 0.05. One representative study of three with similar results is shown.

To further characterise the quality of the memory responses induced by the CpG-adjuvanted EV71 vaccine, we quantified the antibody-secreting B cells in the spleen at 2 weeks after the third vaccination. The frequency of EV71-specific IgG and EV71-specific IgA antibody-secreting cells (ASCs) in the spleen was measured using enzyme-linked immunospot (ELISPOT) assays. Compared with the EV71 group, the EV71 + CpG group showed a significant increase in both EV71-specific IgG ASCs (*p* < 0.05; Fig. 5a,b) and EV71-specific IgA ASCs (*p* < 0.05; Fig. 5c,d). Collectively, these findings showed that CpG used as an adjuvant promoted the expansion of EV71-specific IgG and IgA ASCs in the spleen. Moreover, these data on ASCs in the spleen were consistent with the increased levels of EV71-specific IgGs in the serum (Fig. 1a).

### Cellular immune responses in EV71-immunised mice

**Figure 6:**
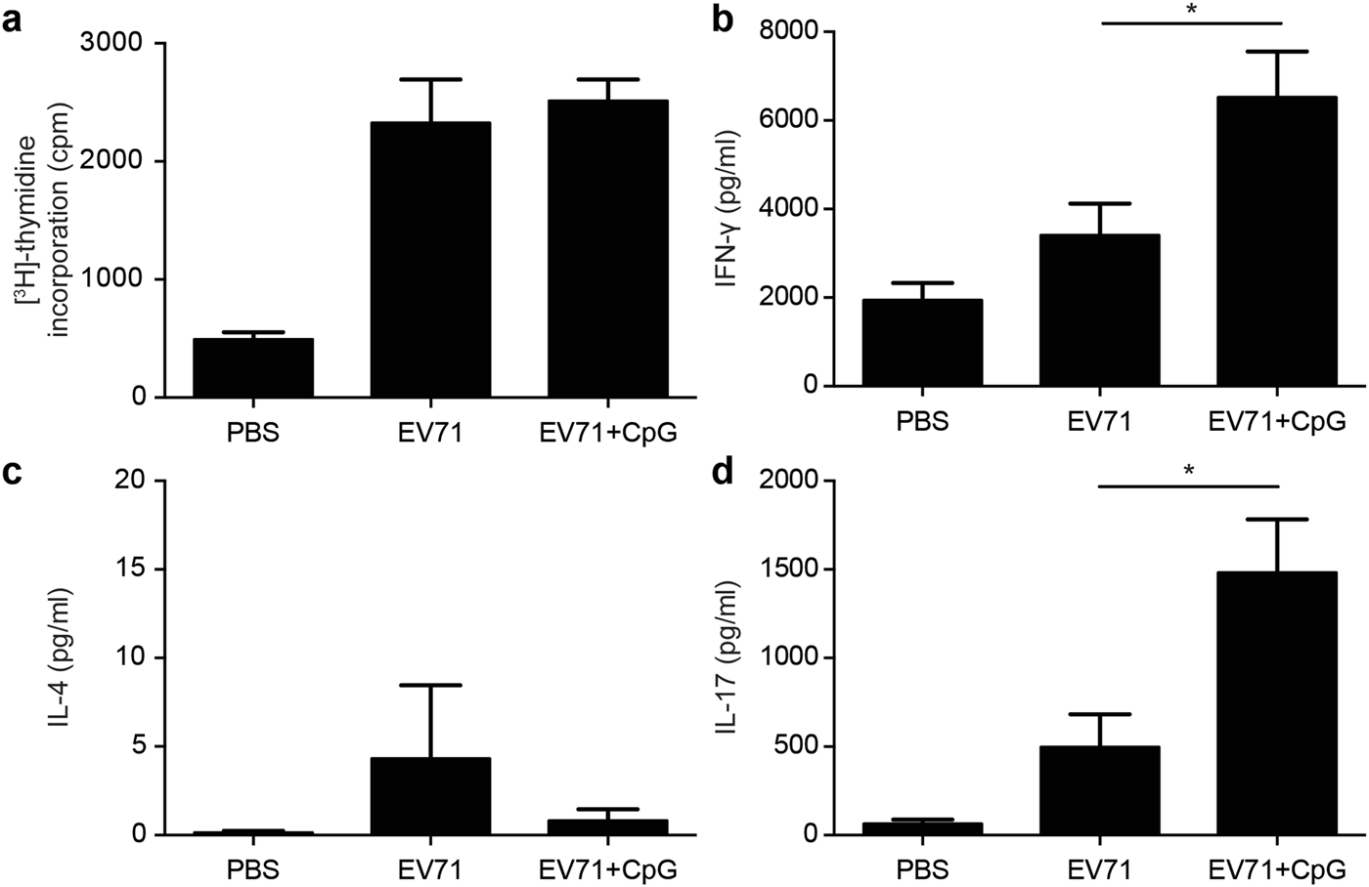
Proliferation and cytokine production of splenocytes derived from immunised mice. Splenocytes from mice immunised with PBS or formalin-inactivated EV71 alone or adjuvanted with CpG were harvested and cultured in medium containing 10 μg/mL heat-inactivated EV71. (**a**) After 5 days in culture, proliferation was measured as [^3^H]-thymidine incorporation. Culture supernatants were analysed after 2 days for IFN-γ (**b**), IL-4 (**c**), and IL-17 (**d**) levels in response to heat-inactivated EV71. Thymidine uptake was determined by harvesting cells and using a scintillation counter to measure the level of incorporation (as counts per minute [cpm]). Values shown are means ± SEMs of six mice in each experimental group; *p < 0.05. One representative study of three with similar results is shown.

Helper T-cell responses play a critical role in generating both humoral and cellular responses. Our results demonstrated enhanced T-cell proliferation and activation in response to the EV71 antigen in mice immunised with EV71 or EV71 + CpG (Fig. 6a). Next, we used ELISA to measure the production of IFN-γ, interleukin (IL)-4, and IL-17 by splenocytes as an indicator of the memory T-cell response. Compared with splenocytes from mice in the PBS group, those from mice vaccinated with EV71 or EV71 + CpG produced significantly higher levels of IFN-γ and IL-17 (Fig. 6b,d) but not IL-4 (Fig. 6c). Notably, the IFN-γ and IL-17 levels in the spleen after EV71 antigen stimulation were higher in the EV71 + CpG group than in the EV71 group (*p* < 0.05; Fig. 6b,d). Overall, CpG used as an adjuvant in an EV71 nasal vaccine could induce increased memory T-cell responses.

### Intranasal (IN) and IM EV71 vaccination in mice induced distinct immune responses

**Figure 7:**
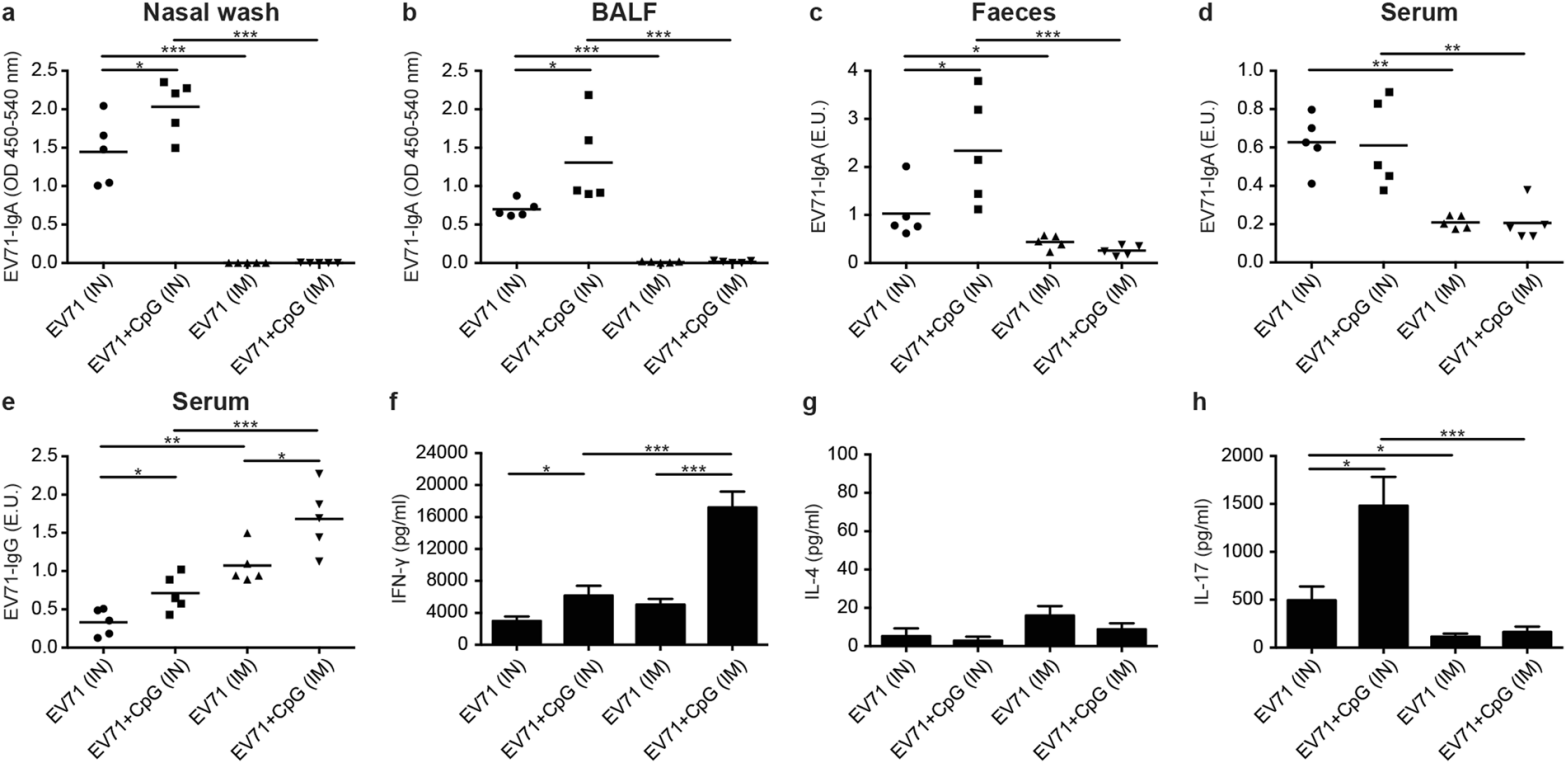
Comparison of the effects of the intranasal (IN) and intramuscular (IM) immunisation routes on EV71-specific antibody titres and cytokine production. Mice were IN or IM immunised with PBS or formalin-inactivated EV71 (5 μg/dose) alone or adjuvanted with CpG (20 μg/dose) three times at 3-week intervals. ELISA was used to measure EV71-specific IgA in the nasal wash (**a**), BALF (**b**), faeces (**c**), and serum (**d**) and EV71-specific IgG in the serum (**e**) of mice at 2 weeks after the third immunisation. Splenocytes from immunised mice were harvested and cultured for 2 days in medium containing 10 μg/mL heat-inactivated EV71, after which culture supernatants were analysed for IFN-γ (**f**), IL-4 (**g**), and IL-17 (**h**) using ELISA. One representative study of two with similar results is shown. Values shown are means ± SEMs of five mice in each experimental group; *p < 0.05, **p < 0.01, ***p < 0.001. BALF: bronchoalveolar lavage fluid.

Mice were immunised with formalin-inactivated EV71 (5 μg/dose) alone or adjuvanted with CpG (20 μg/dose) three times at 3-week intervals through IN inhalation or IM injection. Then, the EV71-specific IgA and IgG titres were measured (Fig. 7) in the nasal wash, BALF, faeces, and serum samples collected 2 weeks after the third immunisation. EV71-specific IgA titres were significantly higher in both the EV71 and EV71 + CpG groups after IN immunisation than after IM immunisation (Fig. 7a–d). The IM vaccination did not induce a marked EV71-specific IgA response, and no facilitating effect of CpG on EV71-specific IgA production was measured. In contrast, IM vaccination potently induced EV71-specific IgG production (Fig. 7e).

We also examined how the IN and IM immunisation routes affected cytokine production. Splenocytes from immunised mice were harvested and cultured for 2 days in the presence of 10 μg/mL heat-inactivated EV71, after which culture supernatants were analysed for IFN-γ, IL-4, and IL-17 using ELISA. Compared with splenocytes from IM-vaccinated mice, splenocytes from mice IN vaccinated with EV71 or EV71 + CpG produced significantly lower IFN-γ (Fig. 7f) and higher IL-17 (Fig. 7h) levels, but produced IL-4 at similar levels (Fig. 7g). Collectively, these results showed that CpG used as adjuvant in the EV71 nasal vaccine induced increased EV71-specific IgA responses and IL-17-secreting T-cell responses in mucosal immunity.

### Protective immunity against a live EV71 challenge

**Figure 8:**
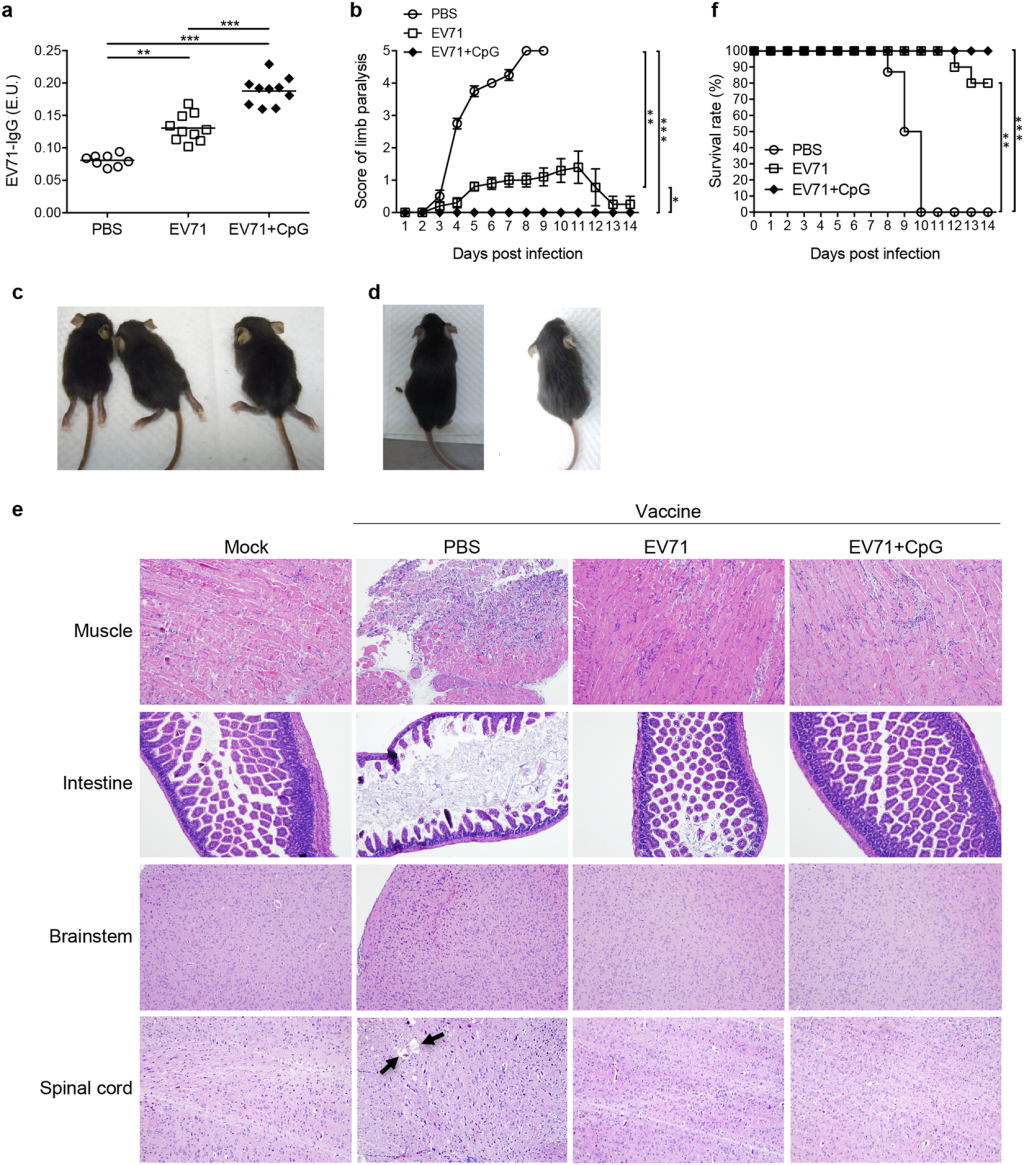
EV71-specific antibody responses, neurological symptoms, pathological changes, and lethality in mice infected with EV71 of the C2 genotype. hSCARB2-Tg mice were intranasally immunised twice with PBS, EV71 (1 μg), or EV71 (1 μg) plus CpG (4 μg) on postnatal days 1 and 2 and were then s.c. challenged with 3 × 10^6^ pfu of the 5746 EV71 strain. (**a**) Serum samples were collected from each mouse on postnatal day 8 before the mouse was s.c. challenged with 3 × 10^6^ pfu of the 5746 EV71 strain, and the collected samples were assayed to determine the titres of EV71-specific IgG. (**b**) CNS disorder-like limb paralysis was scored according to the criteria described in the Methods section. Representative images showing symptoms of (**c**) the PBS-immunised group with limb paralysis and (**d**) protected mice in the EV71 or EV71 + CpG immunised group at day 7 postinfection with EV71 virus. (**e**) Histological examination of EV71-infected mice. The animals were sacrificed in the moribund state, and paraffin sections of the organs, including the muscle, intestine, brainstem, and spinal cord, were stained with H&E. Normal tissues were collected from mice without EV71 challenge as a mock control. (**f**) Daily survival rate, monitored after 8-day-old hSCARB2-Tg mice were s.c. infected with 3 × 10^6^ pfu of the 5746 EV71 strain. Values shown are means ± SEMs of 8–10 mice in each experimental group; *p < 0.05, **p < 0.01, ***p < 0.001.

Human scavenger receptor class B, member 2 transgenic (hSCARB2-Tg) mice represent a useful model for studying the pathogenesis induced by EV71 and for assessing the efficacies of anti-EV71 medications^22^. Here, hSCARB2-Tg mice were IN immunised twice with PBS, EV71 alone, or EV71 + CpG on postnatal days 1 and 2, and serum samples were then collected from the mice on postnatal day 8 before the mice were subcutaneously (s.c.) infected with 3 × 10^6^ pfu of the 5746 EV71 strain (C2 genotype). The samples were assayed for the EV71-specific IgG titre, which was significantly higher in mice IN immunised with EV71 (*p* < 0.01) or EV71 + CpG (*p* < 0.001) than in mice in the PBS group (Fig. 8a). Furthermore, the titre was higher in the EV71 + CpG group than in the EV71 group (*p* < 0.001; Fig. 8a).

Next, limb paralysis resembling that caused by CNS disorders was scored in the mice. After 5–9 days of challenge, hSCARB2-Tg mice immunised with PBS alone exhibited severe limb paralysis (Fig. 8b,c). By comparison, limb paralysis was significantly reduced in hSCARB2-Tg mice immunised with EV71 (*p* < 0.01) or EV71 + CpG (*p* < 0.001), and the mice in the EV71 + CpG group showed no pathological signs (Fig. 8b,d). To verify whether the vaccines could effectively prevent EV71-induced pathogenesis, a histological examination of the muscle, intestine, brainstem, and spinal cord sections was performed (Fig. 8e). The results showed clear and well-organised muscle fibres in uninfected mice (Mock) and vaccinated mice (EV71 or EV71 + CpG group) with infections. The inflammatory cells infiltrated into the muscle tissues, and the destructed structures were observed in the PBS group of EV71-infected mice. In the intestines from vaccinated mice (EV71 or EV71 + CpG group), no damage was observed as compared to the massive destruction of the mucosa and microvilli observed in the PBS group. The brainstem of vaccinated mice from PBS group showed a higher cell density in the upper left area, suggesting hyperplasia of glial cells. The demyelination (arrow) of the spinal cord was observed in the PBS group of EV71-infected mice. In the brainstem and spinal cord from vaccinated mice (EV71 or EV71 + CpG group), no apparent pathology was observed as compared to that in the PBS group.

Lastly, the daily survival rate was monitored in 8-day-old hSCARB2-Tg mice s.c. infected with 3 × 10^6^ pfu of the 5746 EV71 strain (Fig. 8f). On day 13 after infection, survival rates of 80% (*p* < 0.01) and 100% (*p* < 0.001) were observed for the EV71 and EV71 + CpG groups, respectively, whereas no mice survived in the PBS group.

## Discussion

EV71 infection in children is initiated through mucosal surfaces; therefore, mucosal immunisation could represent a promising method for protecting against EV71 infection in humans^23^. In this study, we demonstrated that CpG functioned as an effective mucosal adjuvant when administered IN with an EV71 vaccine in mice. Nasal immunisation induced high titres of anti-EV71 IgA in mucosal areas (nasal wash, BALF, and faeces) and increased anti-EV71 IgAs and IgGs in the serum. Furthermore, the number of EV71-specific ASCs was increased in mice treated with an EV71 vaccine adjuvanted with CpG. The induced antibodies neutralised infection by EV71 of the C2 genotype, and more importantly, the immune reaction resulted in crossprotective immune responses against EV71 of the two other genotypes, B4 and B5. Lastly, administration of a formalin-inactivated whole EV71 vaccine combined with CpG according to a two-dose immunisation protocol conferred protection against infection with a lethal EV71 challenge. Our results confirm that CpG may represent an effective and safe adjuvant for an EV71 mucosal vaccine.

The mucosal surfaces of the respiratory, gastrointestinal, and genitourinary tracts are the primary entry sites of most pathogens, and induction of effective mucosal immunity can substantially reduce infection rates and morbidity. Thus, mucosal immunisation, particularly through the IN route, has attracted considerable interest. Here, an EV71 plus CpG intranasal vaccine induced an EV71-specific IgA immune response at the mucosal surface. EV71-specific IgA was detected in the nasal wash, BALF, and faecal extracts. This could be because distinct mucosal surfaces are interconnected by means of circulating lymphocytes, recognised to constitute the common mucosal immune system^24^, which guide immunisation at one mucosal inductive site to an immune response at a distant mucosal effector site. Among immunogenicity assessments, the detection of ASCs represents a standard measurement of the mucosal immune response. We found that memory B cells and plasma cells secreting EV71-specific IgG and IgA specifically localised in the spleen. The EV71-specific ASC level in the spleen correlated with the titre of EV71-specific antibodies in the serum, nasal wash, BALF, and faeces after IN immunisation. Thus, ASCs can serve as a critical indicator of the subsequent immunological memory at the mucosal surface. Regarding T-cell immunity, the formalin-inactivated EV71 vaccine formulated with CpG and delivered by IN immunisation successfully induced the Th1-type immune response, as shown by the induction of the IgG2a to IgG1 ratio and IFN-γ production. Moreover, a Th17-type immune response was induced, as shown by an increase in IL-17 production. However, no marked changes in IL-4 secretion were observed.

Vaccines introduced IN have been reported to induce stronger mucosal immunity and confer superior protection against mucosal infectious diseases^25,26^ compared with the response elicited by vaccines delivered through systemic routes^27^, such as IM. We investigated the immunomodulatory effects of the CpG adjuvant on the systemic and mucosal immunity induced by the IN and IM EV71 vaccine, which revealed that the adjuvant substantially facilitated EV71-specific IgA and IgG responses and cell-mediated immune responses. The EV71-specific IgA and IL-17 levels were markedly increased in the mucosal tissues of IN immunised mice. In contrast, the EV71-specific IgA and IL-17 levels were extremely low in the mucosal tissue of IM-immunised mice. These data suggest that IL-17 may be involved in the mucosal IgA response in IN immunised mice. Notably, Ahmed *et al*. recently demonstrated that nanoemulsion-based adjuvants, delivered IN together with *Mycobacterium tuberculosis*-specific immunodominant antigens, induced potent mucosal IL-17 T-cell responses^28^. This is consistent with our findings obtained using the EV71 mucosal vaccine. IL-17 was produced mainly by Th17-type lymphocytes. We found that Th1- and Th17-type cytokines were expressed in IN immunised mice, whereas Th1-type cytokines were mainly expressed in IM immunised mice. Although the EV71-specific IgG and IFN-γ production in IN immunised mice was lower than that in IM immunised mice, IN administration can stimulate both mucosal and systemic immunity, whereas IM administration can only stimulate systemic immunity. The advantage of the IN EV71 vaccine is its ability to induce high levels of EV71-specific IgAs to reduce EV71 infection. Furthermore, the vaccination procedure is simpler, more reliable, and cheaper and does not require needles.

EV71 has been divided into several genotypes, including genotypes A, B, and C, and into subgenotypes within genotypes B (B1–B5) and C (C1–C5). In recent years, several large outbreaks of HFMD caused by distinct subgenotypes of EV71 have occurred in East and Southeast Asian countries^4,29^. In Taiwan, different serotypes of EV71 are in circulation; predominant subgenotypes occurred in 1998 (C2), 2000–2002 (B4), 2004–2005 (C4), 2008–2009 (B5), 2010–2011 (C4), and 2011–2012 (B5). In Malaysia from 1997–2000, the cocirculation of four distinct subgenotypes (B3, B4, C1, and C2) has been reported. In South Korea, an EV71 outbreak was reported during 2009, for which the predominant subgenotype was C4. In China, the major subgenotype of EV71 was C4^30^. Thus, for EV71 vaccine development, crossprotection against other EV71 genotypes and subgenotypes is crucial. The inactivated-EV71 mucosal vaccine of the C2 type (1998 strain) induced crossreactive neutralising antibodies against EV71 of the B4 and B5 subgenotypes. We previously demonstrated that formalin-inactivated EV71 of the C2 genotype elicited crossneutralising antibodies against EV71 of the C4 and C5 subgenotypes and, more importantly, the B genotype (B4 and B5) in vaccinated monkey serum^31^. Our results suggested that inactivated EV71 vaccines derived from the subgenotype C2 exhibited broad crossneutralising activity. Based on this finding and on the prior evolution of EV71, we predict that this formalin-inactivated EV71 mucosal vaccine of the C2 subgenotype could prevent future epidemics. Here, neutralising activity against EV71 was not detected in the nasal wash and BALF (data not shown), likely due to the PBS-dilution effect when the nasal wash and BALF were collected; however, the concentrations of EV71-specific IgAs in the nasal wash and BALF were considerably lower than the physiological concentration in the nasal and lung mucosa. Therefore, we speculate that the physiological concentrations of EV71-specific IgAs in the nasal, lung, and intestinal mucosa were sufficient to neutralise EV71 infection.

Adjuvants are widely used owing to their ability to increase the immunogenicity of vaccine antigens. CpG ODN is a potent mucosal immunomodulator for the induction of antigen-specific cell-mediated immunity and humoral immune responses^32,33^. The focus here has mainly been on the direct activation of pDCs, myeloid DCs, and B cells through TLR9-mediated recognition^34^. However, the mechanisms through which CpG functions in IN delivered vaccines require further elucidation. Nasal epithelial cells express various TLRs, including TLR9^35^. In 2015, Qin *et al*. demonstrated that CpG accelerated the delivery of a whole inactivated H9N2 influenza virus through the transepithelial dendrites (TEDs) of DCs in the nasal mucosa^36^. We speculate that DCs expressing SCARB-1 and the P-selectin glycoprotein ligand-1 receptor could increase the probability of capturing EV71 vaccines through TEDs^37,38^, and thus, the EV71-specific response could be initiated. Another potential mechanism could involve the cooperation of various TLRs, which play a leading role after TEDs contact and internalise CpG and EV71. In addition, TLR9 may cooperate with TLR7 in recognising viral nucleic acids associated with EV71.

Lastly, we also examined the efficacy of the EV71 plus CpG IN vaccine in protecting against an EV71 challenge using hSCARB2-Tg mice^22,39^. These transgenic mice have been shown to develop enterovirus-associated diseases mimicking those observed in young children infected with the enterovirus and have been widely used to study enterovirus-related illnesses. We demonstrated that in hSCARB2-Tg mice, IN immunisation with the EV71 vaccine adjuvanted with CpG provided resistance to a subsequent lethal challenge with EV71. This *in vivo* protective function may be conferred by the production of neutralising antibodies and by cellular immune responses.

In summary, our results collectively demonstrated that CpG had the potential to serve as an effective IN adjuvant for the development of vaccines against EV71 infection in humans.

## Methods

### Viruses and vaccines

Three EV71 strains were used in this study: TW/2272/98 (C2 genotype, isolated in 1998), 200307025 (B4 genotype, isolated in 2003), and 20080738 (B5 genotype, isolated in 2008). EV71 viruses were propagated in human RD cells (ATCC no. CCL-136) cultured in minimum essential medium alpha (α-MEM; HyClone) containing 2% foetal bovine serum (FBS; Gibco), at 37 °C in a CO_2_ incubator. The virus was added to RD cells that were 80% confluent, and after 2 days of growth in α-MEM/2% FBS, the supernatant was collected by centrifugation (3200 × *g*, 15 min). The virus-containing supernatant was treated with benzonase for 1 h at 37 °C, and a 300-kDa molecular weight cut-off (MWCO) hollow-fibre filter was used to concentrate the virus-containing supernatant in a tangential-flow filtration system using a transmembrane pressure of ~10–15 psi. After 20–25-fold concentration, the buffer was changed to 50 mM sodium citrate (pH 6.4) containing 0.15 M NaCl through diafiltration. The virus concentrate was purified using an AKTA fast protein liquid chromatography system with a Sephacyl s-400 (26/60) column. PBS was used as the elution buffer, and the flow rate was set at 1.3 mL/min. Fractions containing the virus were pooled, further filtered using a 0.2-μm filter, and concentrated using a 100-kDa MWCO Centricon column. Lastly, the purified EV71 was inactivated by incubating the viruses with 1/4000 formalin for 24 h at 37 °C. Inactivation of the virus was confirmed by TCID_50_ assays. Complete loss of infectivity of the inactivated EV71 viruses used for vaccinating mice was determined by inoculation into RD cells and observation of no cytopathic effects for at least 7 days.

### Immunisation of mice

Six-week-old female BALB/c mice were used to test the effects of CpG (ODN2395, type C; Invivogen, San Diego, CA, USA) as a mucosal adjuvant on the immune response to EV71. For IN immunisation, mice (n = 6 per group) were lightly anaesthetised with isoflurane before being immunised with a 12 μL (6 μL/nostril) prepared vaccine (containing 5 μg of EV71 adjuvanted with or without 20 μg of CpG or PBS alone as a negative control); this was gradually dropped into the nostril to prevent suffocation. To compare the effects of IN and IM immunisation routes, the mice were divided into four groups with five animals per group (n = 5). For IM immunisation, mice were lightly anaesthetised with isoflurane before being injected IM in the quadriceps muscles with 30 μL prepared vaccine (containing 5 μg of EV71 adjuvanted with or without 20 μg of CpG). All mice were vaccinated three times at 3-week intervals, and serum was sampled 2 weeks after the third vaccination, stored at −80 °C, and subsequently used to monitor the immune response. All protocols were reviewed and approved by the Institutional Animal Care and Use Committee (approval no: 20130272) of the College of Medicine, National Taiwan University (Taipei, Taiwan).

To select the antigen dose, we studied the “Dose response of naïve mice to an inactivated EV71 vaccine by intranasal immunisation at different sampling time-points” (unpublished data). We found that mice immunised three times with 5 and 10 μg of inactivated EV71 displayed significantly higher serum IgG titres than mice immunised with 2 μg of inactivated EV71, whereas titres of 5 and 10 μg of inactivated EV71 caused no significant difference. Similar results were observed for the neutralisation titre of serum antibodies. Based on these results, we chose 5 μg of inactivated EV71 as the optimal antigen dose for IN immunisation.

### Nasal wash

The supramaxilla and submaxilla of mice were surgically separated. The nasal cavity was washed with 0.5 mL PBS, and the 0.5-mL wash was then centrifuged at 360 × *g* for 5 min. The supernatant was collected and stored at −80 °C for use in ELISA-based antibody analysis.

### BALF

The trachea of mice was surgically exposed and cannulated, and the entire lung was lavaged three times with 0.5 mL PBS. Next, 0.5 mL BALF was centrifuged at 360 × *g* for 5 min, and the supernatant was collected and stored at −80 °C for antibody analysis using ELISA.

### Faecal extracts

Fresh mouse excrements were collected, adjusted to 100 mg/mL with PBS, and sonicated for 15 min. The dispersed faecal samples were centrifuged at 27,000 × *g* for 10 min at 4 °C, and the supernatant was collected and centrifuged again at 27,000 × *g* for 10 min at 4 °C. The final supernatant was collected as the faecal extract and stored at −80 °C for use in ELISA.

### EV71-specific ELISA

Microplates (Nunc, Rochester, NY, USA) were first coated overnight with 5 μg/mL inactivated EV71 and blocked with Tris-buffered saline containing Tween 20/1% bovine serum albumin (BSA) before adding the serum, nasal-wash, BALF, or faecal extract samples. After a 2-h incubation at room temperature, the plates were washed, followed by the addition of goat anti-mouse IgG horseradish peroxidase (HRP; 1:10000; Bethyl) or anti-mouse IgA HRP (1:5000; Bethyl). For measuring EV71-specific IgG1 or IgG2a, 1 μg/mL biotinylated anti-mouse IgG1 (1:5000; A85-1; Becton, Dickinson and Company, Franklin Lakes, NY, USA) or anti-mouse IgG2a (1:1000; R19-15; Becton, Dickinson and Company) was used; this was followed by incubation for 1 h with HRP-conjugated streptavidin (R&D Systems, Minneapolis, MN, USA). Subsequently, 100 μL of 3,3′,5,5′-tetramethylbenzidine substrate was added. After colour development in the dark at room temperature for 20 min, the reaction was stopped by adding 100 μL of 1 M H_2_SO_4_. The absorbance at 450 and 550 nm was measured using a SpectraMax M5 Multi-Mode Microplate Reader (Molecular Devices, San Jose, CA, USA), and the results are expressed in ELISA units (E.U.): E.U. = (A_sample_ − A_blank_) / (A_positive_ − A_blank_).

### Neutralisation assay

Serum samples were heat-inactivated at 56 °C for 30 min, and then 50 μL of two-fold serially diluted serum samples were mixed with 50 μL of 100-fold TCID_50_ EV71 in 96-well plates and incubated for 1 h at 37 °C. Next, 20,000 RD cells in 100 μL of α-MEM/2% FBS were added into the mixture, and the cytopathic effect was measured after 4 days. The maximal dilution at which no cytopathic effect was detected was regarded as the neutralisation titre.

### ELISPOT

Microplates (Millipore, Billerica, MT, USA) were coated overnight with 10 μg/mL purified EV71 virions and blocked with PBS/3% BSA; then, 800,000 spleen cells in RPMI/10% FBS were added and incubated overnight at 37 °C. The plates were rinsed, and HRP-conjugated goat anti-mouse IgG (1:1000; Bethyl, Montgomery, TX, USA) or IgA (1:1000; Bethyl) was added. The plates were rinsed after a 2-h incubation, and 100 μL of 3-amino-9-ethylcarbazole substrate (Becton, Dickinson and Company) was added per plate. Spots were then developed in the dark at room temperature for 5–10 min. Following the completion of the ELISPOT assay, the plates were air-dried and then scanned and analysed using an ImmunoSpot S6 UV Reader (Cellular Technology Limited, Cleveland, OH, USA). Spots were counted automatically using ImmunoSpot v.6.0 software for each antigen-stimulation condition and the medium negative controls. Spots from four wells containing 3.2 million spleen cells were quantified as one measurement.

### T-cell response analysis

Single-cell suspensions prepared from spleens of immunised mice were stimulated with 10 μg/mL inactivated EV71 in culture medium (RPMI-1640 containing 10% FBS and 2 μg/mL concanavalin A) as the positive control or medium only as the negative control. For cytokine analyses, cells were cultured for 3 days, and supernatants were harvested to detect IFN-γ, IL-4, and IL-17. For proliferation assays, cells were cultured for 5 days and then pulsed with 1 μCi of [^3^H]-thymidine (Amersham Biosciences, Amersham, UK) for 18 h, after which the cells were harvested, and thymidine incorporation was measured using a scintillation counter (TopCount NXT Scintillation and Luminescence Counter; PerkinElmer, Waltham, MA, USA).

### EV71 infection in hSCARB2-Tg mice

hSCARB2-Tg mice were intranasally immunised twice with PBS alone (n = 8), EV71 alone (1 μg; n = 10), or EV71 (1 μg) plus CpG (4 μg; n = 10) on postnatal days 1 and 2 and then were s.c. challenged with 3 × 10^6^ pfu of the 5746 EV71 strain on day 8. The mice were monitored daily for pathological signs, and the severity of CNS syndromes was scored from 0 to 5 according to the following criteria for scoring CNS diseases: 5 = severe front and rear limb paralysis (LP) and no movement; 4 = moderate both rear LP and hesitant movement; 3 = one rear LP with bending of legs; 2 = mild rear limb bending; 1 = slight rear limb bending; 0 = normal movement. LP was defined as rigidness of the legs accompanied by hesitant movement in mice^22^. The Tainan/5746/98 strain (C2 genotype; GenBank: AF304457.1), a clinically isolated strain of EV71, was propagated in Vero cells as previously reported^40^.

### Histopathological staining

Tissues from sacrificed mice were fixed in 10% buffered formalin (Sigma-Aldrich, St. Louis, MO, USA) and embedded in paraffin (Sigma-Aldrich)^22^. Tissue sections (5 μm) were sliced and stained with hematoxylin and eosin (H&E) by the Pathology Core Facility of the National Health Research Institute, Taiwan. All images were acquired at 100× magnification.

### Statistical analysis

Figures were plotted and analysed using GraphPad Prism 6. Student’s *t* tests and one-way analysis of variance were used to compare results between different groups. Differences with *p* values of less than 0.05 were considered statistically significant.

### Data availability

The data associated with the manuscript will be made available only on request. Readers who are interested in requesting the data may contact Dr. Yu-Li Lin (linyuli@ntu.edu.tw).
